# Current News

**Published:** 1898-04

**Authors:** 


### Current News.

### DENTAL SOCIETY OF THE STATE OF NEW YORK.

At the Thirtieth Annual Meeting of the Dental Society of the State of New York, to be held at Albany, May 11 and 12, 1898, papers will be presented by the following members of the profession:

M. W. Foster, M.D., D.D.S., Baltimore, Md.; M. H. Cryer, M.D., D.D.S., Philadelphia, Pa.; J. S. Marshall, M.D., D.D.S., Chicago, Ill.; I. N. Broomell, D.D.S., Philadelphia, Pa.; S. S. Stowell, D.D.S., Pittsfield, Mass.; E. A. Schillinger, D.D.S, Dalton, Mass.; R. Ottolengui, M.D.S., Correspondent; L. C. LeRoy, D.D.S., Committee on Practice.

All members of the profession are cordially invited to attend and participate in the exercises commemorative of the occasion.

H. J. Burkhart,
*President.*

C. S. Butler, Buffalo, N. Y.,
*Secretary.*

### PENNSYLVANIA STATE BOARD OF DENTAL EXAMINERS.

THE Board of Dental Examiners for the State of Pennsylvania will hold two examinations this spring of candidates for license to practise dentistry in this State. The first commencing April 12, simultaneously in Philadelphia and Pittsburg. The second commencing June 14 in Philadelphia. Each examination will take four days. In making application to the Dental Council at Harrisburg for a license, those desiring to come before this board in April will please state in which city they prefer to be examined.

J. C. GREEN,
*Secretary.*

### KANSAS STATE DENTAL ASSOCIATION.

THE Twenty-seventh Annual Meeting of the Kansas State Dental Association will be held at Topeka, May 10, 11, and 12, 1898. An interesting programme is being prepared. Members of the profession are invited to attend and participate in proceedings.

EDWARD BUMGARDNER,
*Secretary.*

### ILLINOIS STATE DENTAL SOCIETY.

THE Thirty-fourth Annual Meeting of the Illinois State Dental Society will be held at Springfield, May 10 to 13, 1898. Dentists practising in the State of Illinois who are not members of the Society, and dentists of other States, are cordially invited to attend. Hotels and railroads will make the usual reduction. A large attendance is anticipated.

A. H. PECK,
*Secretary.*

92 STATE STREET, CHICAGO.

### IOWA STATE DENTAL SOCIETY.

THE annual meeting of the Iowa State Dental Society will be held in Des Moines, May 3, 4, 5, and 6, 1898.

WILLIAM GILMORE CLARK,
*Secretary.*

